# Exploring the Activity of Fungal Phenalenone Derivatives as Potential CK2 Inhibitors Using Computational Methods

**DOI:** 10.3390/jof8050443

**Published:** 2022-04-24

**Authors:** Sabrin R. M. Ibrahim, Alaa A. Bagalagel, Reem M. Diri, Ahmad O. Noor, Hussain T. Bakhsh, Yosra A. Muhammad, Gamal A. Mohamed, Abdelsattar M. Omar

**Affiliations:** 1 Department of Chemistry, Preparatory Year Program, Batterjee Medical College, Jeddah 21442, Saudi Arabia; 2 Department of Pharmacognosy, Faculty of Pharmacy, Assiut University, Assiut 71526, Egypt; 3 Department of Pharmacy Practice, Faculty of Pharmacy, King Abdulaziz University, Jeddah 21589, Saudi Arabia; abagalagel@kau.edu.sa (A.A.B.); rdiri@kau.edu.sa (R.M.D.); aonoor@kau.edu.sa (A.O.N.); htbakhsh@kau.edu.sa (H.T.B.); 4 Department of Pharmaceutical Chemistry, Faculty of Pharmacy, King Abdulaziz University, Jeddah 21589, Saudi Arabia; yosra.muhammad2017@gmail.com (Y.A.M.); asmansour@kau.edu.sa (A.M.O.); 5 Center for Artificial Intelligence in Precision Medicines, King Abdulaziz University, Jeddah 21589, Saudi Arabia; 6 Department of Natural Products and Alternative Medicine, Faculty of Pharmacy, King Abdulaziz University, Jeddah 21589, Saudi Arabia; gahussein@kau.edu.sa; 7 Department of Pharmaceutical Chemistry, Faculty of Pharmacy, Al-Azhar University, Cairo 11884, Egypt

**Keywords:** phenalenones, fungi, cancer, casein kinase, CK2 inhibitor, molecular docking

## Abstract

Cancer represents one of the most prevalent causes of global death. CK2 (casein kinase 2) activation boosted cancer proliferation and progression. Therefore, CK2 inhibition can have a crucial role in prohibiting cancer progression and enhancing apoptosis. Fungi have gained vast interest as a wealthy pool of anticancer metabolites that could particularly target various cancer progression-linked signaling pathways. Phenalenones are a unique class of secondary metabolites that possess diverse bioactivities. In the current work, the CK2 inhibitory capacity of 33 fungal phenalenones was explored using computational studies. After evaluating the usefulness of the compounds as enzyme inhibitors by ADMET prediction, the compounds were prepared for molecular docking in the CK2-α1 crystal structure (PDB: 7BU4). Molecular dynamic simulation was performed on the top two scoring compounds to evaluate their binding affinity and protein stability through a simulated physiological environment. Compound **19** had a superior binding affinity to the co-crystallized ligand (**Y49**). The improved affinity can be attributed to the fact that the aliphatic chain makes additional contact with Asp120 in a pocket distant from the active site.

## 1. Introduction

Cancer is a complicated illness that is featured by uncontrolled cell proliferation and the development of tumors that may remarkably extend to the entire organ or other organs systemically [1]. It represents one of the most global deadly diseases, particularly in western countries. It was estimated that in 2020, ~ 9.9 million people have died due to cancer [2]. Its current treatment strategies include γ-radiation, chemotherapy, suicide gene therapy, or immunotherapy, which are fundamentally mediated by promoting apoptosis [3]. Chemotherapy is the most efficacious method for metastatic tumors treatment. However, the cancer cell’s multidrug resistance, high cost, and untoward effects of these drugs represent the main holdbacks to the success of chemotherapeutic treatment [4]. Therefore, searching for sources of safe and effective treatment has become a crucial research area.

Casein kinase 2 (CK2) is one of the first discovered Ser/Thr kinases [5,6,7] that is involved in many cell processes from gene expression and protein synthesis to cell growth, proliferation, and differentiation [8]. The well-studied tetrameric form of this kinase is composed of two catalytic alpha proteins, CK2α and/or CK2α`, that differ only in their C-termini [9,10,11], and two regulatory CK2β proteins that are responsible for substrate specificity [11,12,13]. It has minimal substrate specificity; therefore, it is able to phosphorylate a large number of proteins involved in multiple signal transduction pathways [14]. This enzyme is ubiquitously expressed [7] and constitutively active in cells; hence, its activity is not relied on extracellular stimuli [5,15,16]. Many reports have found that the overexpression of CK2 in many cancer types leads to inhibiting apoptosis and promoting cancer cell survival [5,8]. It is clear that its downregulation revered cancer progression and enhanced apoptosis [17], suggesting that CK2 can be considered a valuable target for anticancer agents [8,18].

Natural metabolites and their derivatives have long been established as a wealthy source for the discovery of novel anticancer drugs [19]. It was reported that ~5% of the anticancer drugs approved by the FDA are unmodified natural metabolites, and ~50% of the approved drugs are either chemically-modified natural metabolites or synthesized relied on natural metabolites structures [20].

It is noteworthy that different metabolites belonging to various classes, such as flavonoids, coumarins, anthraquinones, pyrazolotriazines, polyhalogenated benzimidazoles, and benzotriazoles, have been known as CK2 inhibitors [21]. Additionally, CIGB-300 and CX-4945 are CK2 inhibitors that are already in human trials as anticancer drugs. CX-4945 (Silmitasertib) has been designated by the FDA for treating cholangiocarcinoma, and many clinical investigations are ongoing with it versus different types of cancers [22]. Furthermore, CIGB-300 is under investigation for cervical cancers [23].

Fungi are eukaryotic micro-organisms that inhabit nearly all kinds of environments in nature, where they play a fundamental role in maintaining eco-balance [24,25,26,27,28]. Fungi have drawn immense research attention since their cultivation, isolation, characterization, and purification demonstrated the existence of a vast number of metabolites with unique and diversified chemical classes and bioactivities [29,30,31,32,33,34]. Many reports revealed the characterization of diverse classes of fungal metabolites with CK2 inhibitory potential [35,36,37].

**Figure 1:**
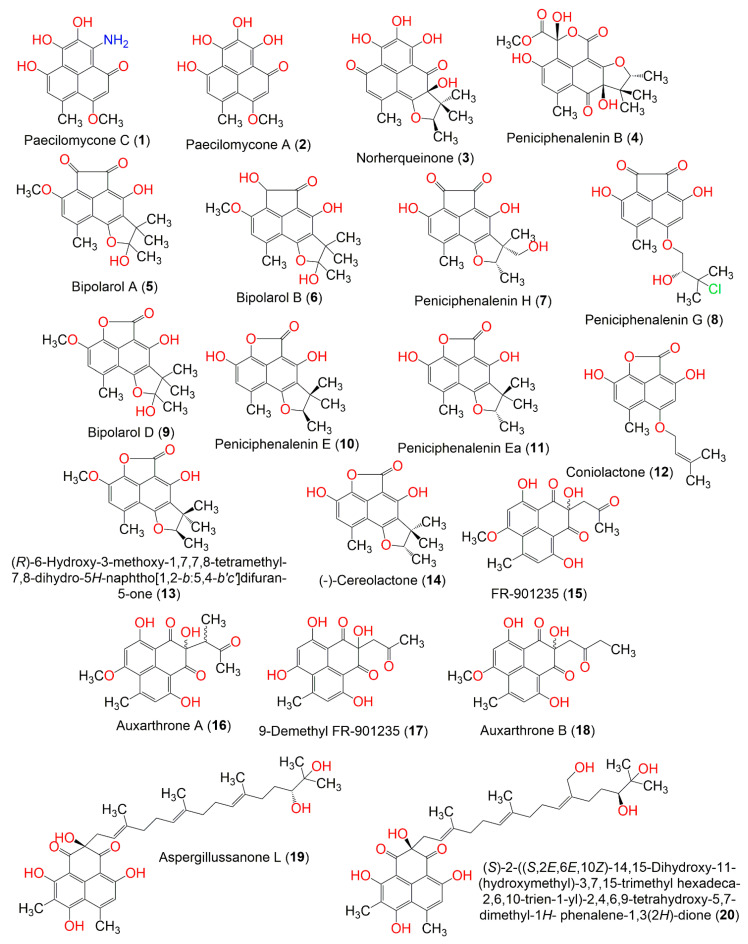
Structures of phenalenone derivatives **1**–**20**.

**Figure 2:**
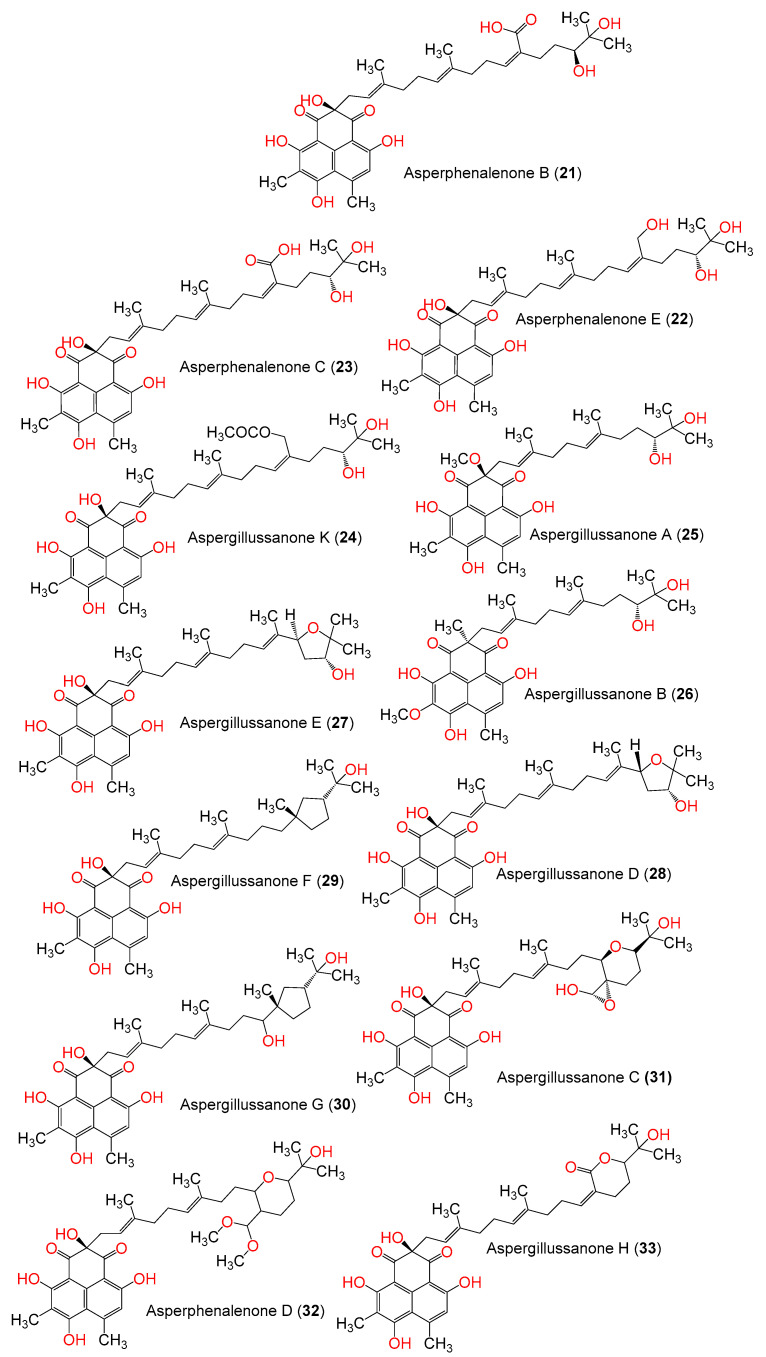
Structures of phenalenone derivatives **21**–**33**.

Phenalenones are a unique class of secondary metabolites, having a fused three-ring system [38]. They are produced by higher plants and fungi [39]. They are known as phytoalexins, which are biosynthesized by the plant in response to an external threat, such as mechanical injury or pathogenic infections [40]. Fungal phenalenones have immense structural diversity, such as hetero- and homo-dimerization and high degrees of nitrogenation and oxygenation, as well as being complexed with metals, incorporating with additional carbon frameworks, or isoprene unit by the formation of either a linear ether (e.g., **8** and **12**) or a trimethyl-hydrofuran (e.g., **3**–**7**, **9**–**11**, **13**, and **14**) moiety. It has been reported to possess various bioactivities, including antimicrobial, anti-plasmodial, anticancer, antidiabetic, antioxidant, and cytotoxic effects [40]. These metabolites exhibit cytotoxic capacities towards various cancer cell lines; however, there are limited or lacking studies that explore the mechanism of their anticancer properties. It is noteworthy that there is no available report on their CK2 inhibitory potential. The molecular docking-based virtual screening process, along with increasing the data about the structure of CK2 alone or in complex with various inhibitors, could become of particular relevance in the discovery of new lead compounds as CK2 inhibitors [41,42]. In continuation of our interest in exploring the bioactivities of fungal metabolites, the present work focuses on the in silico assessment of CK2 inhibitory capacity of 33 phenalenone derivatives reported from various fungal sources (Table 1, Figure 1 and Figure 2).

Computer-aided drug design/discovery (CADD) are useful tools for screening and identifying drug-like molecules in silico and thereby reducing the number of compounds to be tested experimentally. Several software and programs are used to filter and generate a group of compounds based on specified criteria, predict their physicochemical properties, predict suitable targets, and evaluate the binding affinity of the compounds to the predicted targets. One of the drug design and discovery approaches is structure-based drug design (SBDD). This approach relies on the knowledge of the 3D structure of the targets of interest, and it includes two common methods: molecular docking and molecular dynamic simulation. Molecular docking evaluates how tight the compound binds the target, as determined by the predicted binding affinity, while molecular dynamic (MD) simulations assess the behavior of the ligand-protein complex in terms of binding interactions and 3D conformation in aqueous conditions to mimic the physiological environment [43]. Several CADD methods and tools are used for investigating the phenalenone derivatives.

## 2. Results and Discussion

### 2.1. AI (Artificial Intelligence)-Based Target Prediction for Phenalenone Derivatives

Choosing the appropriate target to investigate the inhibitory potential of these phenalenone derivatives relied on performing ligand-based-in silico target prediction [38]. The prediction webserver, SuperPred was the tool of choice to perform ATC (anatomical-therapeutic chemical) code and predict the potential targets for the investigated phenalenones [57]. After analyzing the results of the predicted target proteins (for example, Cathepsin D, mineralocorticoid receptor, and thyroid hormone receptor-α), the kinase CK2α was selected for the study due to having a high percent of probability and model accuracy (Table 2). After selecting the target and proper crystal structure, the listed phenalenones were docked in the protein crystal structure, after which the docking method was validated by redocking the co-crystallized ligand. Prediction of ADMET properties of the listed metabolites in silico and MD (molecular-dynamic) simulation for the two metabolites with the highest docking scores were followed.

### 2.2. Ligands and Protein Preparation and Molecular Docking

Target identification filtered out > 100 phenalenone derivatives. These derivatives were prepared for docking, where their energy-minimized 3D structures were generated, and all possible ionization and tautomeric states were created.

For docking, the human CK2α1 crystal structure (PDB ID: 7BU4) was chosen for the study due to the structural similarity of the co-crystallized ligand (**Y49**) to the selected phenalenones. The **Y49** (4-(6-aminocarbonyl-8-oxidanylidene-9-phenyl-7*H*-purin-2-yl)benzoic acid) is made of three aromatic rings: a purine ring with two phenyl moieties attached to it, and polar groups present at the rings, with polar groups at different positions. The selected phenalenones have a nucleus made of three-fused rings substituted with multiple OH and carbonyl groups. Based on their structures, both the phenalenones and **Y49** were expected to have a similar 3D conformation in the binding pocket. The PDB file of the 7BU4 crystal structure was downloaded from the protein data bank (PDB) [58], which was then prepared and minimized using Schrodinger’s Protein Preparation wizard [59,60,61].

The docking process started by generating a grid box around the binding site of the co-crystallized ligand to locate the pocket where the docking of the compounds occurs. A receptor-Grid-Generation tool in Maestro [62] was utilized for that purpose.

**Figure 3:**
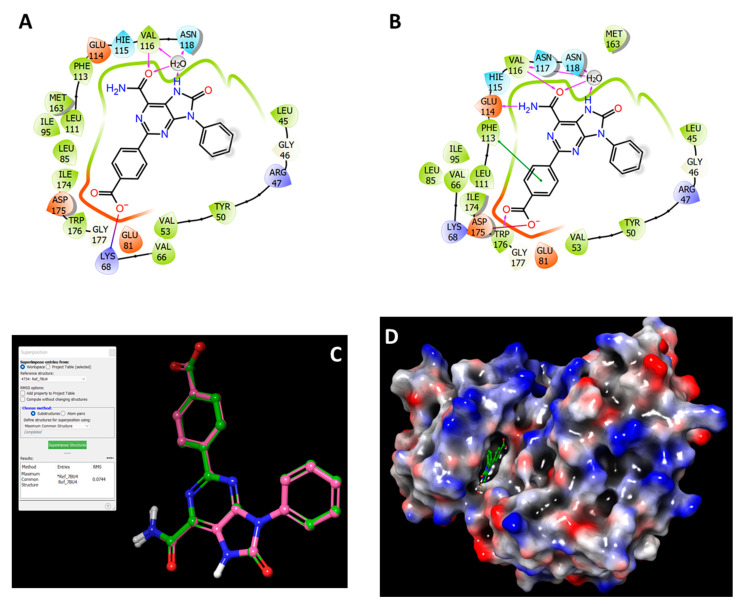
Re-docking of the co-crystallized ligand to validate the docking method. The figure showed the 2D view of the binding interactions of the reference ligand **Y49** complexed with CK2α1; (**A**) after minimization of the crystal structure 7BU4, and (**B**) after re-docking of ligand **Y49** into the CK2α1 crystal structure. (**C**) 3D structure of the re-docked **Y49** (pink) superimposed on the co-crystallized Y49 (green). (**D**) Molecular surface display with electrostatic potential color scheme for CK2α1 complexed with ligand **Y49** after re-docking.

Re-docking of the co-crystallized ligand, 4-(6-aminocarbonyl-8-oxidanylidene-9-phenyl-7*H*-purin-2-yl)benzoic acid (PDB: **Y49**), was performed to evaluate the docking method. The re-docked reference had an identical pose (Figure 3C) and binding interactions to the co-crystallized structure (Figure 3A,B). For both, the backbone of Val116 is H-bonded with the oxygen of the amide moiety, and the purinone nitrogen interacts through the water bridge with Val116, Asn118, as well as with the amide oxygen. On the opposite side of the molecule, the carboxylate makes an ionic interaction with the side chain of the adjacent Lys68 (Figure 3A,B). The RMSD (root-mean-square deviation) of the re-docked ligand was minimal, with a value of 0.0744 Å, indicating the docking method is valid (Figure 3C). The molecular surface display in Figure 3D shows the re-docked reference **Y49** occupying the binding pocket of the crystal structure.

After docking validation, docking the 3D structures of the > 100 phenalenones that proceeded from the target prediction using the extra-precision (XP) mode was followed. The docking produced derivatives that are ranked based on their score and approximated the free energy of binding; the more negative the value, the stronger the binding. Different docking scores were generated, including the gscore (best for ranking different compounds), emodel (best for ranking conformers), and XP gscore. Glide uses emodel scoring to select the best poses of the docked compounds; then, it ranks the best poses based on the given gscores. XP gscore ranks the poses generated by the Glide XP mode. In general, Glide uses gscore to sort and rank the docked compounds. The 33 derivatives listed in Table 3 are the ones with gscores close to or better than the gscore of the reference **Y49** (−9.049 kcal/mol), with the top two compounds, **19** and **31**, scoring −12.878 and −12.521 kcal/mol, respectively.

**Figure 4:**
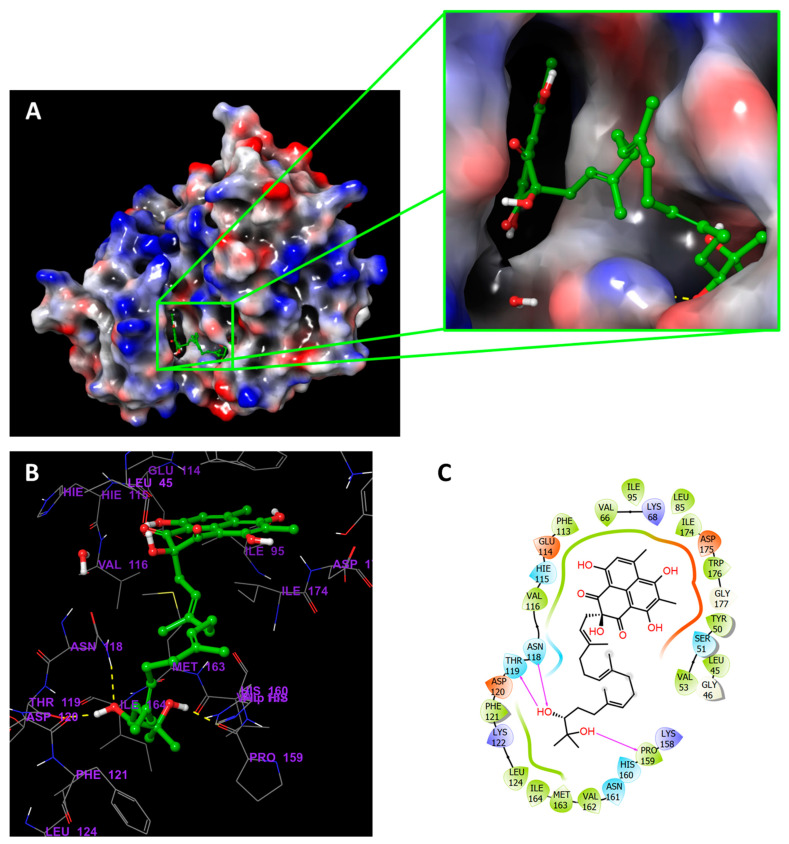
CK2α1 in complex with **19**. (**A**) Molecular surface display with electrostatic potential color scheme for CK2α1-**19** complex and the close-up view presented. (**B**) 3D presumed binding mode of **19** in the binding site of CK2 (PDB:7BU4). Compound **19** is displayed as green ball-and-sticks. The amino acids in the binding site are shown as grey sticks, and hydrogen bonds are represented in yellow dotted lines. (**C**) 2D depiction of the ligand-protein interactions.

**Figure 5:**
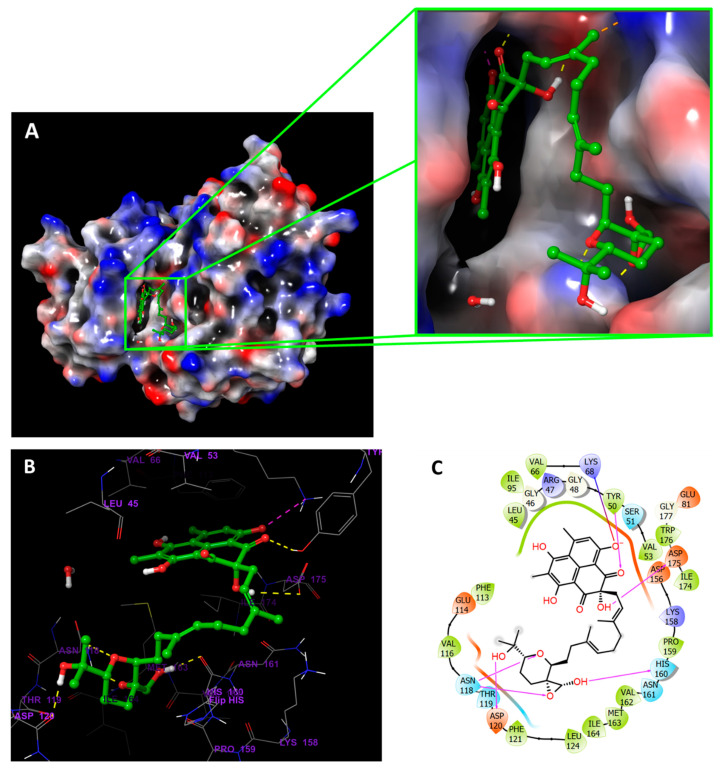
CK2α1 in complex with **31**. (**A**) Molecular surface display with electrostatic potential color scheme for CK2α1-**31** complex and the close-up view presented. (**B**) 3D presumed binding mode of **31** in the binding site of CK2 (PDB:7BU4). Compound **31** is displayed as green ball-and-sticks. The amino acids in the binding site are shown as grey sticks, and hydrogen bonds are represented in yellow dotted lines. (**C**) 2D depiction of the ligand-protein interactions.

**Figure 6:**
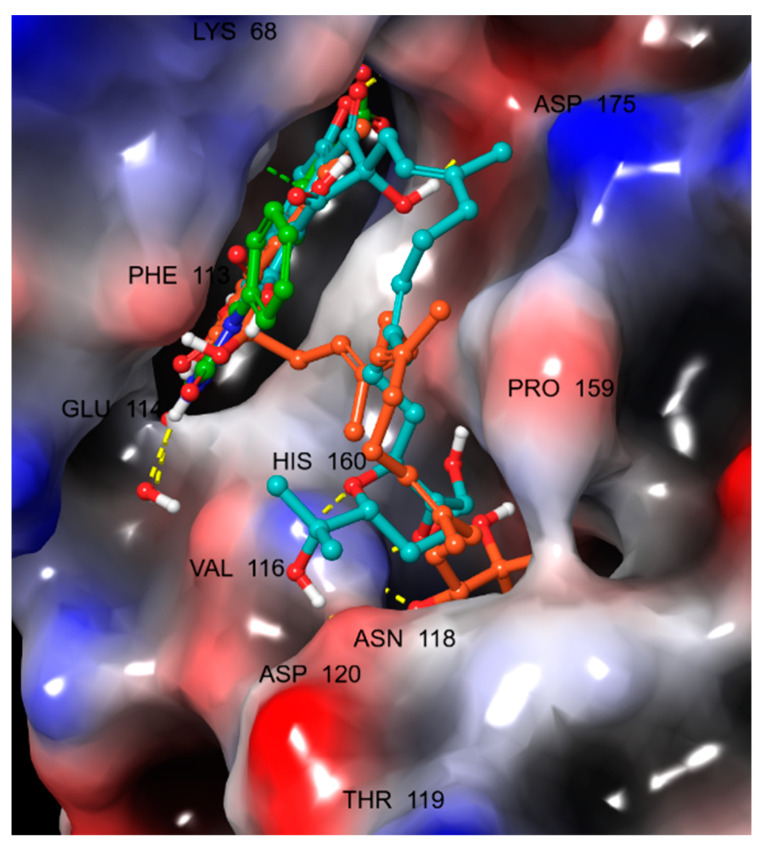
Molecular display with electrostatic potential color scheme for CK2α1 (PDB: 7B4U) showing **19** (orange) and **31** (cyan) superimposed on **Y49** (green) inside the binding pocket.

Compound **19**, in addition to interacting directly with Val116 and Lys68 in the protein’s binding pocket like the reference ligand, had a long chain that extended along the surface of the protein allowing the terminal (R,6*E*,10*E*,14*E*)-2,6,10,14-tetramethylhexadeca-6,10,14-triene-2,3-diol group to bind a distant binding pocket (Figure 4). Besides, compound **31** seems to have similar interactions with the protein; however, the tetrahydropyran at the end of the aliphatic chain did not occupy the distant pocket like **19** and remained exposed to the solvent (Figure 5). Figure 6 showed compounds **19** and **31** simultaneously superimposed on the reference **Y49** inside the binding pocket in the molecular surface display.

### 2.3. In Silico ADMET Properties of Selected Ligands

The drug-likeness and ADMET properties of the processed compounds were predicted using Maestro’s QikProp Schrodinger module in terms of absorption, distribution, metabolism, excretion, and toxicity, among others [63]. The module can quickly and reliably predict many physicochemical properties and other descriptors, such as the number of possible metabolites and number of reactive functional groups, in order to detect and filter compounds that can be problematic during the late stages of drug discovery and development. Therefore, it can eliminate unnecessary tests and experiments that will ultimately fail in clinical trials [64]. The ADMET prediction evaluates the usefulness of the examined compounds by describing and determining their drug-likeness, physicochemical properties, and expected toxicity profiles. Several descriptors were predicted for these derivatives, and most of the predicted values of ADMET descriptors fell within the recommended range. The predicted ADMET properties and descriptors are presented in Table 4.

### 2.4. Molecular Dynamic (MD) Simulation

**Figure 7:**
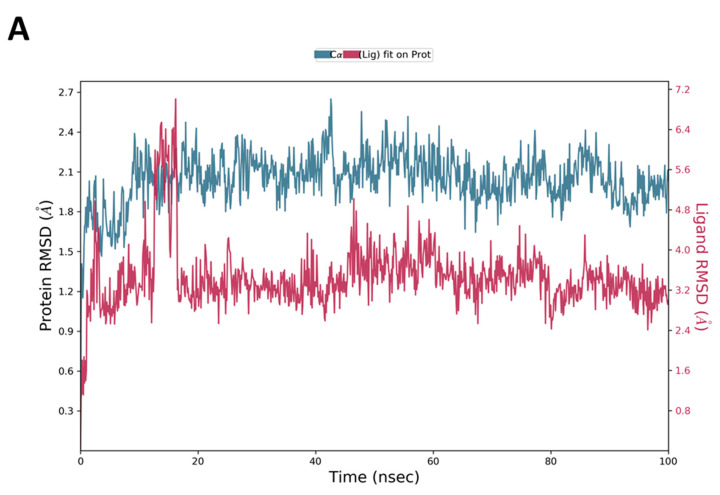

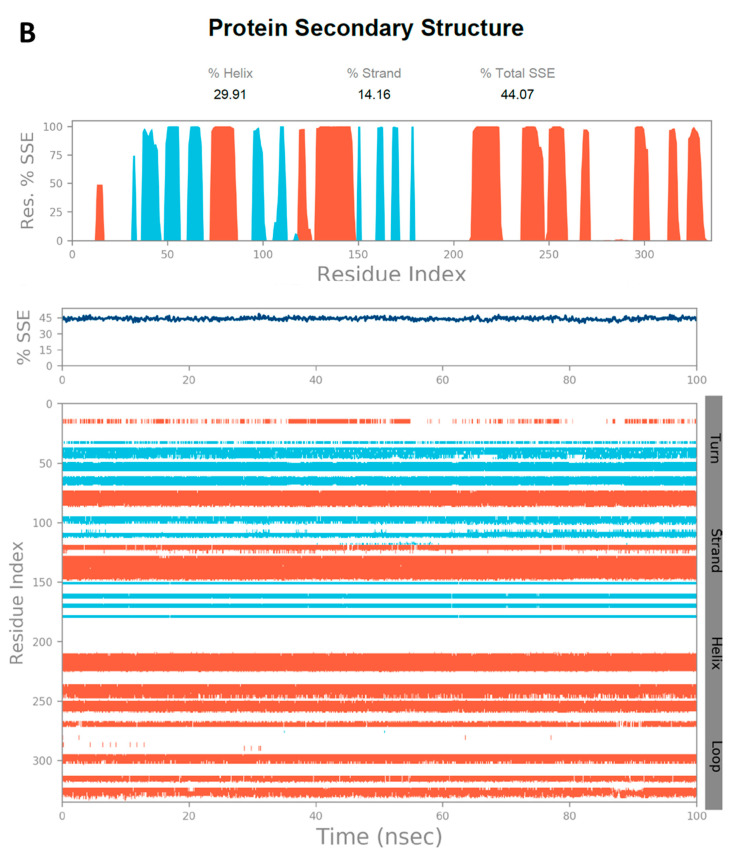
(**A**) The RMSD plot was obtained for the reference **Y49** complexed with CK2α1 protein (PDB-ID:7BU4). The simulation time (100 ns) reaffirmed the stability of the complex with no significant changes in the protein structure. (**B**) Stability of the secondary structure of CK2α1 protein (PDB ID: 7BU4) was evaluated by monitoring its SSE distribution (top plot), SSE composition (middle plot), and SEE assignment (bottom plot) over the 100 ns of MD simulation when complexed with **Y49**.

**Figure 8:**
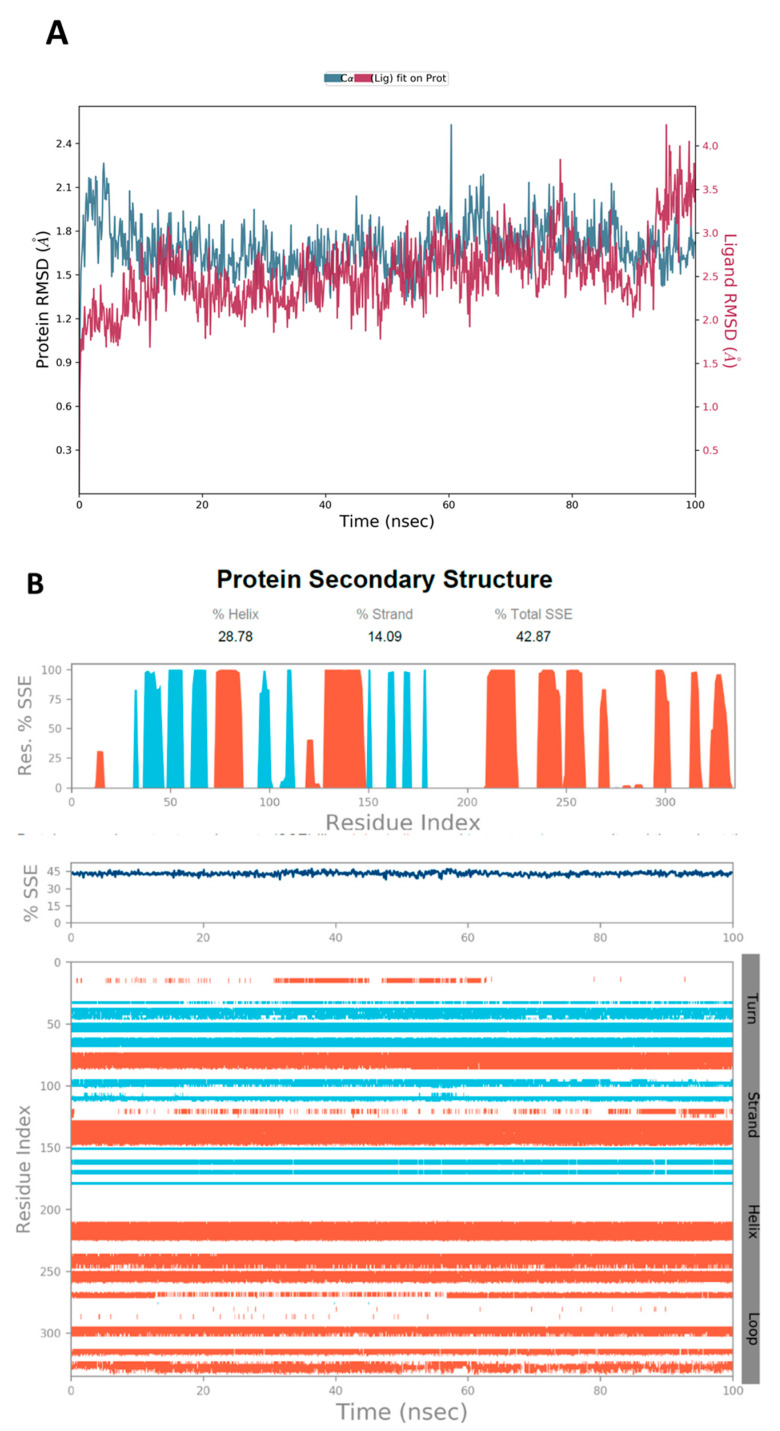
(**A**) The RMSD plot was obtained for compound **19** complexed with CK2α1 protein (PDB-ID:7BU4). The simulation time (100 ns) reaffirmed the stability of the complex with no significant changes in the protein structure. (**B**) Stability of the secondary structure of CK2α1 protein (PDB ID: 7BU4) was evaluated by monitoring its SSE distribution (top plot), SSE composition (middle plot), and SEE assignment (bottom plot) over the 100 ns of MD simulation when complexed with **19**.

**Figure 9:**
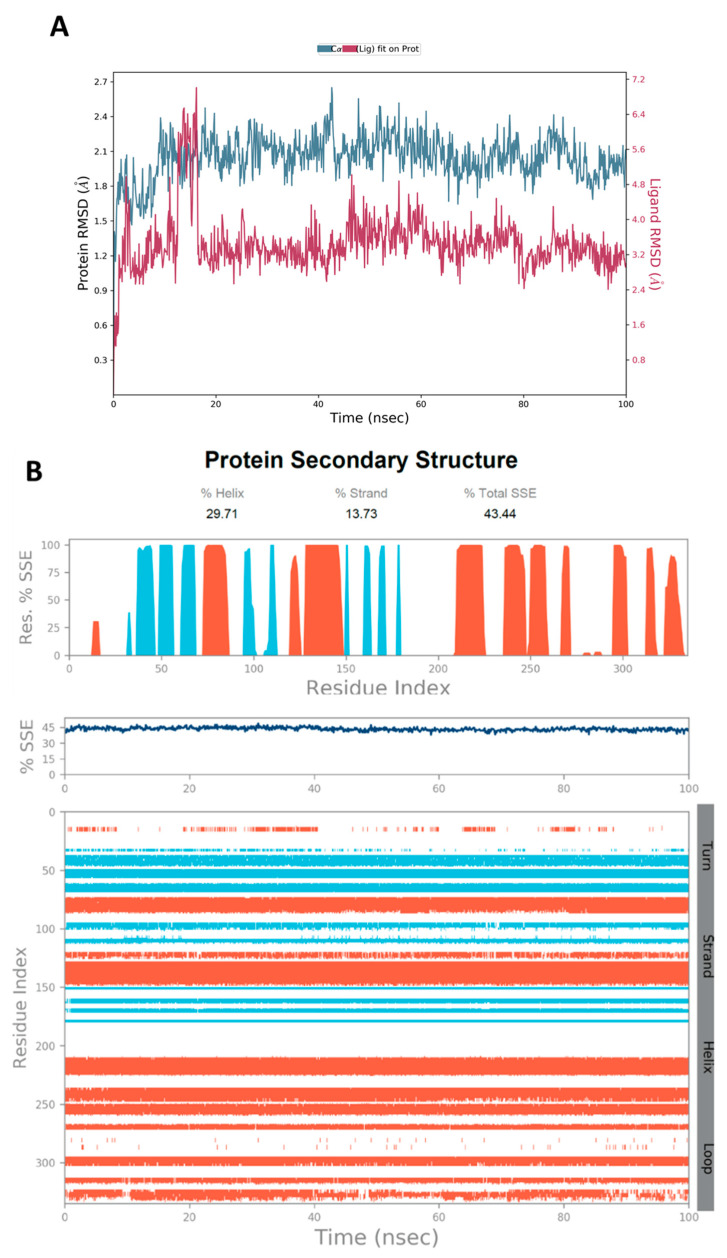
(**A**) Obtained RMSD plot for compound **31** complexed with CK2α1 protein (PDB-ID:7BU4). The simulation time (100 ns) reaffirmed the stability of the complex with no significant changes in the protein structure. (**B**) Stability of the secondary structure of CK2α1 protein (PDB ID: 7BU4) was evaluated by monitoring its SSE distribution (top plot), SSE composition (middle plot), and SEE assignment (bottom plot) over the 100 ns of MD simulation when complexed with **31**.

The MD simulations are performed using Desmond software [65,66] to simulate the aqueous physiological environment and assess the changes in protein conformation and binding affinity during the simulation time compared to the original affinity and confirmation of the crystal structure [67]. Only the two top-scoring compounds from the docking study, i.e., compounds **19** and **31** along with reference **Y49**, were analyzed by MD. The root mean square deviation (RMSD) is a calculated value that compares the poses of investigated compounds to that of the co-crystalized ligand [43]. RMSD plots of the selected compounds complexed with the CK2α1 measure the average change in the positions of the atoms of the protein and ligand inside the binding pocket at the end of the simulation period (100 ns) compared to their starting positions before the simulation at 0 ns. The RMSD plot of **Y49** showed that both the protein and the reference **Y49** were stable, and the observed fluctuations were insignificant since they were within the acceptable range of 1–3 Å (Figure 7A). For compound **19**, the RMSD of the protein and **19** laid over each other, indicating increased binding affinity of **19** to the protein and stability of the CK2α1-**19** complex. Additionally, the fluctuation seen for both over the 100 ns was within the range as well (Figure 8A). A similar RMSD pattern was observed for **31** and CK2α1 complex, despite the sudden, non-significant fluctuation of **31** at around 90 ns, which is potentially a result of the compound adjusting its pose in the pocket (Figure 9A). When calculating the RMSD for the compounds, it is not uncommon to observe fluctuation in the plot for some time at the beginning of the simulation, as observed in Figure 7A, Figure 8A, and Figure 9A within the first 20 ns of the run. This expected fluctuation happens as the compound keeps adjusting its conformation inside the pocket to assume a pose that has the least free energy.

The secondary structure of the CK2α1 protein (PDB ID: 7BU4) was also evaluated throughout the simulation while it was complexed with each ligand. Figure 7B represented the protein evaluation while it was complexed with ligand **Y49**. The top plot showed the distribution of the SSE (α-helices and β-sheets) with the protein represented by the residue index. The middle plot summarized the SSE composition for each trajectory frame throughout the simulation, while the bottom plot monitored each residue and its SSE assignment over the simulation time. Both plots indicated that the overall %SSE of the protein was maintained, and each SSE was stable over the course of the simulation. Comparable results were obtained when **19** (Figure 8B) and **31** (Figure 9B) were complexed with the protein.

**Figure 10:**
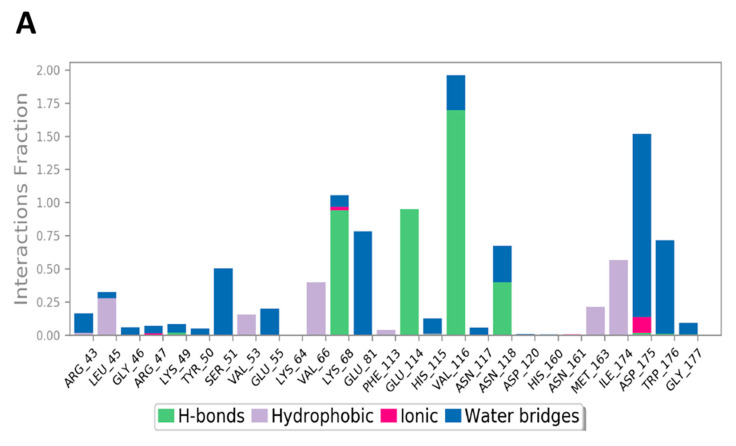

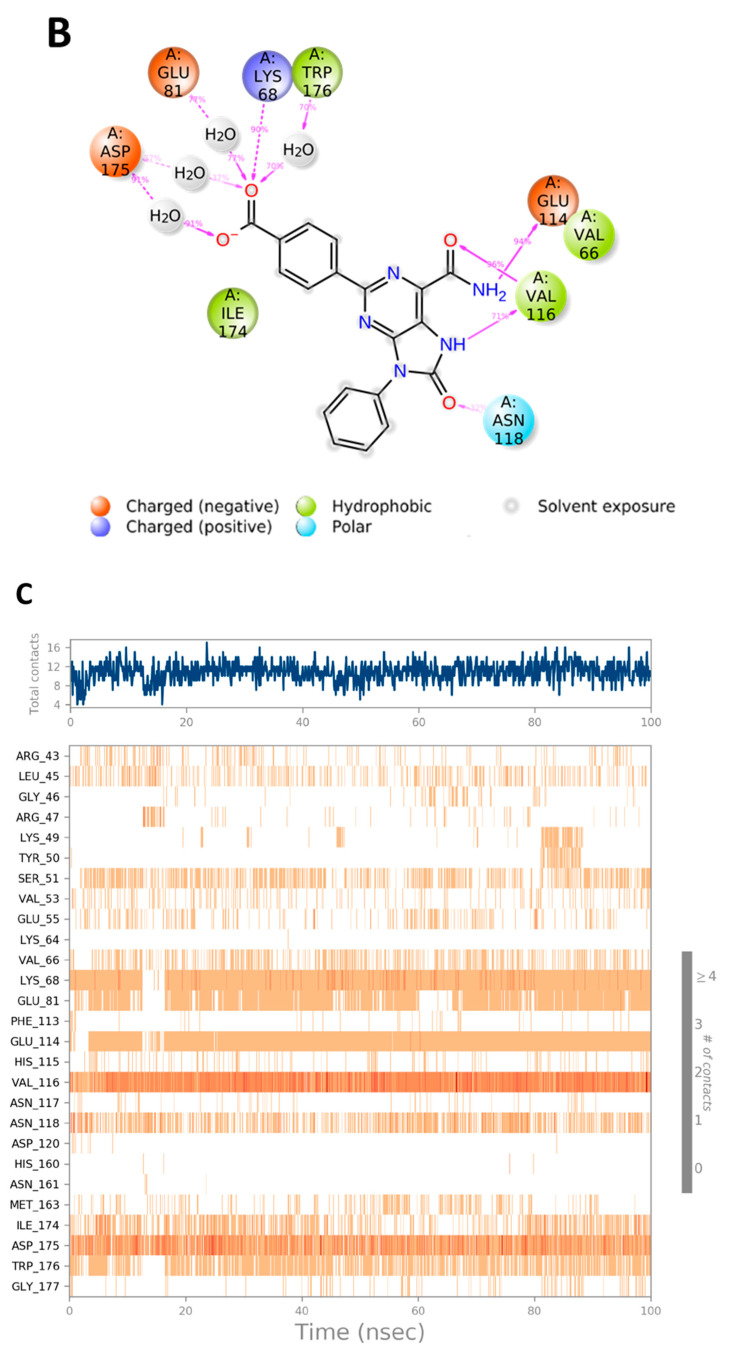
(**A**) Stacked bar graph for CK2 interactions with reference **Y49** throughout the simulation. (**B**) Schematic diagram shows the detailed 2D atomic interactions of **Y49** with CK2 that occurred > 30% of the simulation time in the selected trajectory. (**C**) A timeline representation of CK2-**Y49** interactions presented in (**A**). The top panel presents the total number of specific interactions the protein made with the ligand over the course of the trajectory. The bottom panel presents the residues interacting with the ligand in each trajectory frame. The dark orange color indicates more than one specific interaction is made between some residues and the ligand.

The MD study also evaluated the binding interactions of a protein-ligand complex. For ligand **Y49**, the interactions between the **Y49** and protein are presented in Figure 10A; the interaction types are color-coded. The stacked bar chart is normalized over the course of the trajectory: for example, a value of 0.8 suggested that the specific interaction was maintained during 80% of the simulation time. Values over 1.0 are possible and indicate that some protein residue may make multiple interactions of the same subtype with the ligand. As indicated in Figure 10A, Val116 made direct H-bonding as well as through water bridges with **Y49** and had a normalized value of ~1.9. The value >1 represented the combined value of >1 type of interaction, and it indicated that these interactions were maintained for ~190% of the simulation time. The other key interactions were with Glu114, Asn118, Lys68, and Asp175, having values of ~0.9, ~0.7, ~1.1, and ~1.5, respectively. Figure 10B showed only the interactions between **Y49** and the protein that occurred ≥ 30% of the simulation time. Figure 10C displayed the total specific interactions between ligand **Y49** and the protein (top plot), whilst the bottom panel demonstrated the protein residues that interacted with the ligand at each time point. As mentioned earlier, Val116 made > 1 interaction with the ligand, which was represented by the dark orange color in the plot throughout the trajectory. Other residues: Lys86, Glu114, and Asp175, also made specific interactions with the ligand.

**Figure 11:**
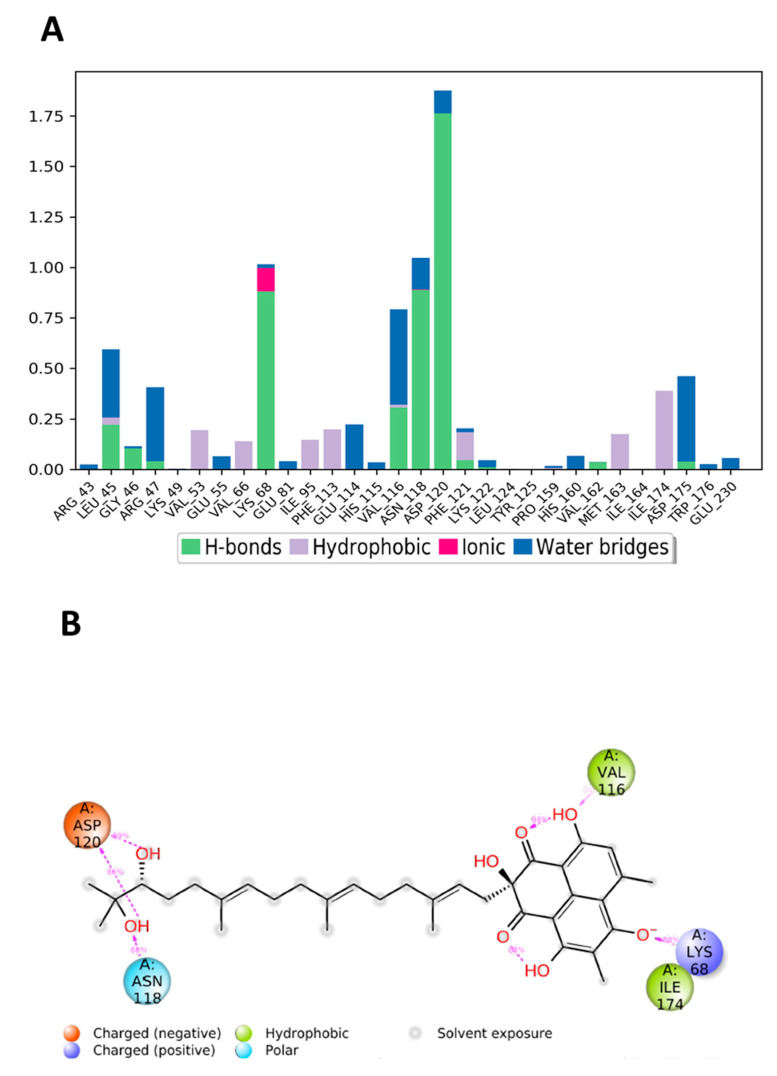

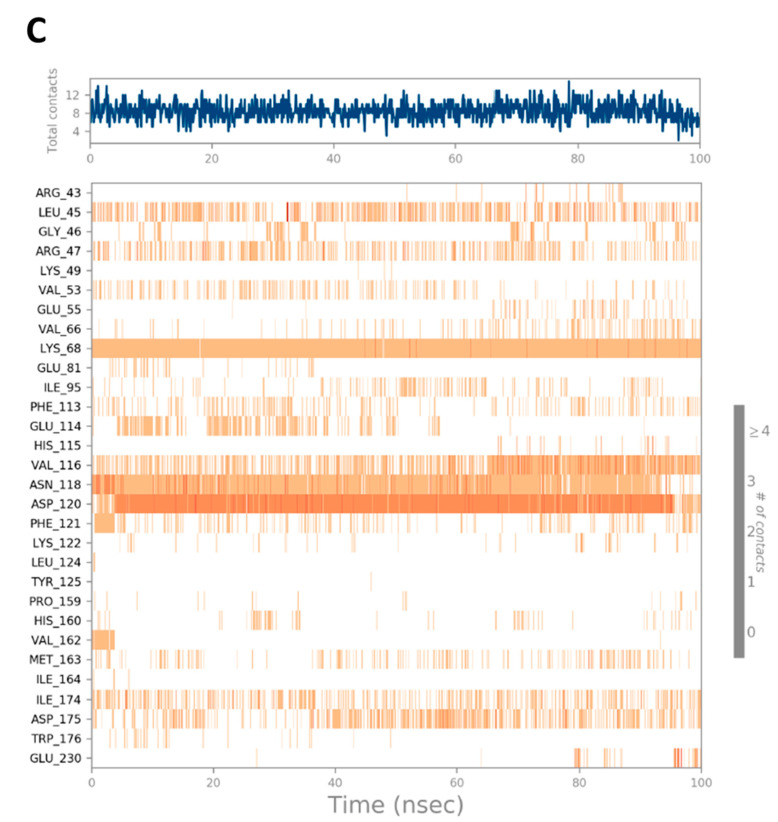
(**A**) CK2 interactions with compound **19** throughout the simulation. (**B**) Schematic diagram shows the detailed 2D atomic interactions of **19** with CK2 that occurred > 30% of the simulation time in the selected trajectory. (**C**) A timeline representation of CK2-**19** interactions presented in (**A**). The top panel presents the total number of specific interactions the protein made with the ligand over the course of the trajectory. The bottom panel presents residues interacting with the ligand in each trajectory frame. The dark orange color indicates that more than one specific interaction is made between some residues and the ligand.

Figure 11 shows the amino acid residues of the protein binding pocket that interacted with compound **19**. The fused ring system of **19** made similar interactions with the pocket residues as **Y49**, where Val116 and Lys86 interacted through the H-bond with the ring system (Figure 11B). As previously seen in the molecular surface display (Figure 4A), the extended aliphatic chain occupied a distant pocket and created new interaction points between the two OH groups at the end of the chain and Asp120, where they occurred > 85% during the simulation (Figure 11B). This interaction was not present with **Y49** (Figure 3B,D). It might be safe to assume that the enhanced binding affinity and stability of the complex were due to this new interaction with Asp120, which can also be inferred from the RMSD plot (Figure 8A).

**Figure 12:**
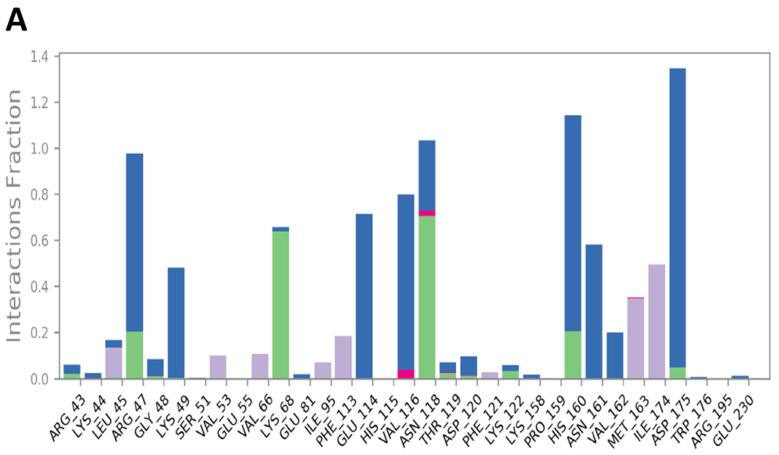

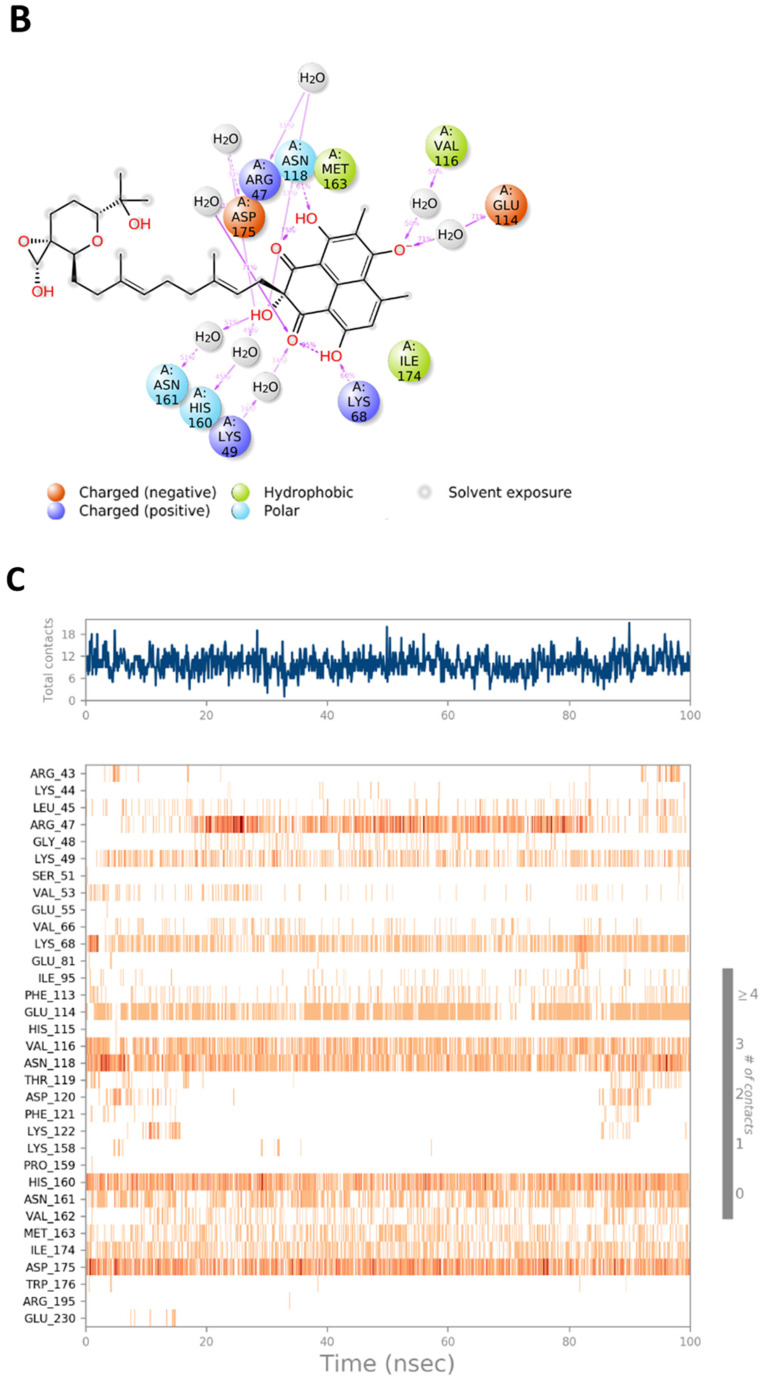
(**A**) CK2 interactions with compound **31** throughout the simulation. (**B**) Schematic diagram shows the detailed 2D atomic interactions of **31** with CK2 that occurred > 30% of the simulation time in the selected trajectory. (**C**) A timeline representation of CK2-**31** interactions presented in (**A**). The top panel presents the total number of specific interactions the protein made with the ligand over the course of the trajectory. The bottom panel presents residues interacting with the ligand in each trajectory frame. The dark orange color indicates more than one specific interaction is made between some residues and the ligand.

**Figure 13:**
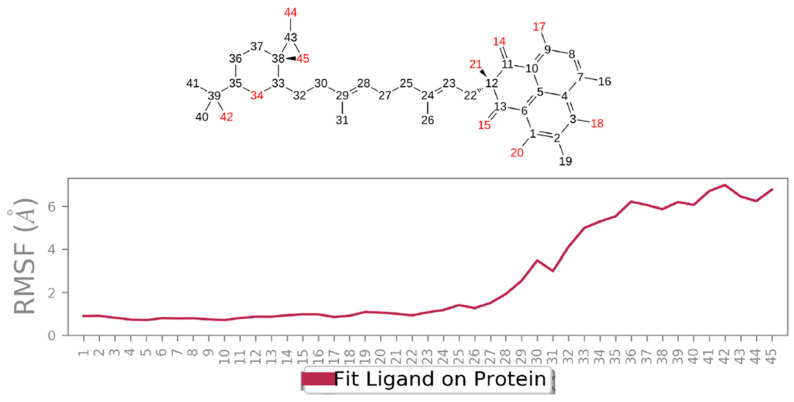
Ligand RMSF shows the fluctuations of **31** broken down by atom as represented by the compound’s 2D structure. It provides ideas on how ligand atoms interact with the protein and their entropic role in the binding event. The ‘Fit Ligand on Protein’ line presents the ligand fluctuations, with respect to the protein. The CK2α1-**31** complex was first aligned on the protein backbone, and then the ligand RMSF was measured on the atoms of the ligand.

Compound **31** also created new interacting points with amino acids in the main binding pocket: the chiral OH of the fused ring system made a strong H-bonding with His160 and Asn161 through bridging water molecules with a value of ~1.18 and 0.6, respectively. An enhanced interaction with Arg47 was observed as well (~0.98) (Figure 12B). The tetrahydropyran ring at the end of the aliphatic chain of **31** also extended along the protein surface but did not occupy the distant pocket, as did compound **19** (Figure 5A). The reason for that might be the fact that the chain in compound **31** is 6-carbon shorter than that of **19**, so the group could not reach the second pocket. Another explanation could be the large size of the substituted tetrahydropyran hindered the group from occupying the pocket. Additionally, there is a high probability that the fluctuation observed in the RMSD of **31** towards the end of the simulation time (Figure 9A) might be a result of the inability of this group to bind to the second pocket. The L-RMSF (ligand-root-mean-square fluctuation) represents how the atoms of the ligand interact with the protein and the changes in the positions of the ligand atoms. As seen in the L-RMSF plot for compound **31** (Figure 13), the positions of atoms 29–45 were dramatically changed because of the free rotation around the aliphatic chain, which in turn decreased the interaction between this part of the molecule with the protein and was reflected by > 3 Å fluctuation in the RMSF plot. The time-depended representation of the CK2α1-**31** interactions showed that residues Arg47, His160, and Asp175 were the ones making specific interactions with the ligand, as indicated by the darker color in the plot (Figure 12C).

It was also noticed that the reference ligand, **Y49,** as well as the compounds **19** and **31,** all interacted with Asn118 through H-bonding; however, the contact points are different. While the residue interacted with **Y49** at the purine carbonyl oxygen (Figure 10B), it acted as an H-bond donor to the OH at the end of the aliphatic chain of **19** as well as to the OH at the fused ring system of **19** and **31** (Figure 11B and Figure 12B). The reason for the different contact points of Asn118 with the compounds was probably attributed to the 3D conformation of the compounds inside the binding pocket, which is affected by the nature of the substitutions on the nucleus. Each compound assumed a pose that had the lowest possible free energy when interacting with the pocket’s residues. Therefore, that pose with the different substitutions from one compound to the other created the unique binding interactions with the pocket amino acids.

## 3. Conclusions

CK2 was related to many human illnesses, not only cancer, but also multiple sclerosis, cardiac hypertrophy, neurodegenerative and inflammatory disorders, cystic fibrosis, and virus infections [68]. It is noteworthy that the CK2 role is best recognized and investigated in cancer, where CK2 is almost positively upregulated, resulting in tumor progression because of its role in regulating nearly all the essential processes for developing apoptosis suppression [5,69]. It was reported that cancer cells rely on CK2 high levels compared to normal cells, which supports that the CK2 inhibitors can have a crucial contribution to cancer therapy development [5]. Recently, several compounds have been discovered and optimized via rational drug design approaches. Various structure-based drug design (SBDD) tools have been utilized for CK2 drug discovery for predicting possible compound and target interactions and their affinity. Various classes of natural metabolites, such as anthraquinones, benzoimidazoles, coumarins, pyrazolotriazines, and flavonoids, are recognized as CK2 inhibitors [70,71]. Fungal phenalenones are a fascinating class of fungal metabolites with diverse bioactivities that could be lead metabolites for drug discovery. With the aid of computational methods, i.e., ADMET, docking, and MD simulation, compounds **19** and **31** were identified as promising drug-like phenalenone derivatives that have better binding interactions and protein stability in a simulated aqueous physiological environment. The current work highlights the usefulness of these metabolites as lead for anticancer discovery. One of the important issues that require attentiveness is that several mechanistic studies are directed to the in silico methods because they provide information that cannot be obtained by other models and are less time-consuming. However, in vivo and in vitro investigations are warranted to strengthen the findings of in silico studies and provide opportunities for observing other mechanisms of the anticancer potential of these metabolites.

## 4. Materials and Methods

### 4.1. Target Prediction

The webserver SuperPred is a knowledge-based method that uses machine learning models for ATC code and target prediction of investigated compounds [57]. The machine learning model uses logistic regression and Morgan fingerprints of length 2048. The drugs approved by the WHO are classified by a drug classification system that connects the drugs’ chemical properties and therapeutic properties and indications, where each classification is given a code called an Anatomical Therapeutic Chemical (ATC) code. Therefore, if a drug has more than one therapeutic indication, it is given an ATC code for each indication. The WHO has 6300 approved drugs that are linked to over 600,000 targets. Based on the hypothesis that compounds that have similar physiochemical properties exhibit similar biological effects, the webserver translates a user-defined compound into a structural fingerprint and compares this fingerprint to that of the WHO-approved drugs. When similarity is found, the webserver predicts the ATC code, the possible therapeutic target(s), and the putative therapeutic indication(s) for that compound. In other words, if an investigated compound is structurally similar to an approved drug, the compound is predicted to have biological activity on all possible targets of that drug. After targets are predicted, a probability score and a model accuracy score are reported. The probability represents the chance that the investigated compound will bind to a specific predicted target. The model accuracy reflects the performance accuracy of the used machine-learning model when predicting that specific target for the compound since the model performance differs between targets [57,72]. The targets and ATC codes for a library of investigated compounds were predicted using the SuperPred tool. The compounds that did not have the common target(s) of interest were excluded from further analysis. The ones sharing the common target(s) were advanced for the docking and further studies.

### 4.2. Preparation of PDB Structures

#### 4.2.1. Ligand Preparation

Phenalenone derivatives were processed and prepared for docking using Schrodinger’s LigPrep tool [40]. The 2D structures were converted to 3D and energy-minimized using OPLS3 force-field. After adding hydrogens, all possible ionization states and tautomeric forms were created at pH of 7.0 ± 0.2 by Epik; desalt option was also chosen. H-bonds were optimized by predicting the pKa of ionizable groups suing PROPKA [73].

#### 4.2.2. Protein Preparation

CK2 crystal structure (PDB: 7BU4) was prepared using the Protein Preparation Wizard, added hydrogens to residues, changed covalent bonds to metal ions to zero-order, and created disulfide bonds. Water molecules > 5 Å from protein residues were deleted. Using Epik, the protonation state of residues was generated, and the formal charge on metal ions was adjusted. After removing the extra protein subunits of multi-subunit proteins and additional ligands, processing of the protein was refined by predicting the pKa of ionizable residues using PROPKA [73], and water molecules > 3 Å (not involved in water bridge) were removed. Finally, restrained minimization of the protein was applied using the OPLS4 force field.

### 4.3. Grid Generation and Docking

A grid box was generated around the co-crystallized ligand **Y49** in the protein crystal structure (PDB: 7BU4) binding site using Glide’s Receptor-Grid-Generation tool [62]. Inside this box is where the docking of the phenalenone compounds was performed. The non-polar atoms were set for the VdW radii scaling factor by 1.0, and the partial charge cut-off was 0.25. Docking was then performed by the Schrodinger suite “Ligand Docking” tool [62,74]. The selected docking protocol was standard precision (SP), and the ligand sampling method was flexible. All other settings were default. Re-docking of the co-crystallized ligand (PDB: **Y49**) was performed to evaluate the docking method and docking of the investigated phenalenones followed.

### 4.4. ADMET Properties Prediction

The processed compounds were subjected to ADMET prediction using the QikProp-module of the Schrodinger suite [63]. The descriptors: molecular weight (mol_MW), drug-likeness (#Stars), dipole moment (dipole), total solvent accessible surface area (SASA), number of hydrogen bond donors and acceptors (donorHB and acceptHB), predicted octanol-water partitioning (QPlogPo/w), predicted aqueous solubility (QPlogS), estimated binding to human serum albumin (QPlogKhsa), number of the possible metabolites (# metab), predicted blood-brain partitioning (QPlogBB), percentage of human oral absorption, predicted IC_50_ for inhibiting HERG-K^+^ channels (QPogHERG), central nervous system activity (CNS), and number of reactive functional groups present (#rtvFG), were predicted for these derivatives. The predicted values are compared to the recommended range derived from values determined/observed for 95% of known drugs.

### 4.5. MD Simulation

MD simulations were performed using Desmond software in the Schrodinger suite [65,66]. The protein-ligand complexes of interest were retrieved from the docking results where the force field was OPLS4. The complexes were tuned through the “System-Builder” tool to generate the solvated system for simulation. The solvent model was set as TIP3P, the selected box shape was orthorhombic, and the box dimensions were 10 Å. Na ions were added to neutralize the system. The simulation parameters were set up in the Molecular Dynamic tool, where the protein-ligand complexes were evaluated at pH 7.0 ± 0.2 over the 100 ns simulation time. The ensemble class was set as NPT in order to maintain the temperature and pressure constant during the run at 300 K and 1.01325 bar, respectively. After running the MD simulation, the generated results were analyzed.

## Figures and Tables

**Table 1:** List of phenalenone derivatives and their fungal source.

Compound Name	Fungal Source	Ref.
Paecilomycone C (**1**)	*Paecilomyces gunnii*	[44]
Paecilomycone A (**2**)	*Paecilomyces gunnii*	[44]
Norherqueinone (**3**)	*Penicillium* sp. G1071	[45]
Peniciphenalenin B (**4**)	*Penicillium* sp. ZZ901	[46]
Bipolarol A (**5**)	*Lophiostoma bipolare* BCC25910	[47]
Bipolarol B (**6**)	*Lophiostoma bipolare* BCC25910	[47]
Peniciphenalenin H (**7**)	*Pleosporales* sp. HDN1811400	[48]
Peniciphenalenin G (**8**)	*Pleosporales* sp. HDN1811400	[48]
Bipolarol D (**9**)	*Lophiostoma bipolare* BCC25910	[47]
Peniciphenalenin E (**10**)	*Penicillium* sp. ZZ901	[46]
Peniciphenalenin Ea (**11**)	*Penicillium* sp. ZZ901	[46]
Coniolactone (**12**)	*Chrysosporium lobatum* TM-237-S5	[49]
(R)-6-Hydroxy-3-methoxy-1,7,7,8-tetramethyl-7,8-dihydro-5H-naphtho[1,2-b:5,4-b’c’]difuran-5-one (**13**)	*Trypethelium eluteriae*	[50]
(-)-Cereolactone (**14**)	*Penicillium herquei* PSU-RSPG93	[51]
FR-901235 (**15**)	*Auxarthron pseudauxarthron* TTI-0363	[52]
Auxarthrone A (**16**)	*Auxarthron pseudauxarthron* TTI-0363	[52]
9-Demethyl FR-901235 (**17**)	*Talaromyces stipitatus*	[53]
Auxarthrone B (**18**)	*Auxarthron pseudauxarthron* TTI-0363	[52]
Aspergillussanone L (**19**)	*Aspergillus* sp.	[54]
(*S*)-2-((*S*,2*E*,6*E*,10*Z*)-14,15-Dihydroxy-11-(hydroxymethyl)-3,7,15-trimethylhexadeca-2,6,10-trien-1-yl)-2,4,6,9-tetrahydroxy-5,7-dimethyl-1*H*-phenalene-1,3(2*H*)-dione (**20**)	*Aspergillus* sp.	[54]
Asperphenalenone B (**21**)	*Aspergillus* sp. CPCC 400735	[55]
Asperphenalenone E (**22**)	*Aspergillus* sp. CPCC 400735	[55]
Asperphenalenone C (**23**)	*Aspergillus* sp. CPCC 400735	[55]
Aspergillussanone K (**24**)	*Aspergillus* sp.	[54]
Aspergillussanone A (**25**)	*Aspergillus* sp. PSU-RSPG185	[56]
Aspergillussanone B (**26**)	*Aspergillus* sp. PSU-RSPG185	[56]
Aspergillussanone E (**27**)	*Aspergillus* sp.	[54]
Aspergillussanone D (**28**)	*Aspergillus* sp.	[54]
Aspergillussanone F (**29**)	*Aspergillus* sp.	[54]
Aspergillussanone G (**30**)	*Aspergillus* sp.	[54]
Aspergillussanone C (**31**)	*Aspergillus* sp.	[54]
Asperphenalenone D (**32**)	*Aspergillus* sp. CPCC 400735	[55]
Aspergillussanone H (**33**)	*Aspergillus* sp.	[54]

**Table 2:** Prediction of target probability and model accuracy for phenalenone derivatives against CK2 using SuperPred target prediction webserver.

Compounds	Probability *	Model Accuracy **
**1**	61%	99%
**2**	65%	99%
**3**	89%	99%
**4**	88%	99%
**5**	83%	99%
**6**	88%	99%
**7**	83%	99%
**8**	50%	99%
**9**	85%	99%
**10**	94%	99%
**11**	94%	99%
**12**	91%	99%
**13**	92.9%	99%
**14**	94%	99%
**15**	75.7%	99%
**16**	75%	99%
**17**	76%	99%
**18**	76%	99%
**19**	75.7%	99%
**20**	75%	99%
**21**	79%	99%
**22**	75%	99%
**23**	79%	99%
**24**	80%	99%
**25**	76%	99%
**26**	76%	99%
**27**	82%	99%
**28**	82%	99%
**29**	81%	99%
**30**	86%	99%
**31**	83.39%	99.23%
**32**	83.6%	99%
**33**	88%	99%

* The probability of the test compound binding to a specific target, as determined by the respective target machine learning model. ** The accuracy of the performance of the prediction model displaying the 10-fold cross-validation score of the respective logistic regression model, as the model performance varied between different targets.

**Table 3:** In silico docking results of phenalenone derivatives with CK2α1 (PDB: 7BU4).

Compounds	Docking Score	Glide GScore	Glide Emodel	XP GScore
**19**	−12.181	−12.878	−84.318	−12.878
**31**	−10.976	−12.521	−100.255	−12.521
**20**	−10.654	−12.303	−102.849	−12.303
**23**	−10.798	−11.495	−92.818	−11.495
**26**	−8.802	−10.538	−88.527	−10.538
**33**	−9.828	−10.526	−80.067	−10.526
**22**	−9.712	−10.409	−85.63	−10.409
**27**	−9.706	−10.403	−81.743	−10.403
**24**	−8.859	−10.403	−96.868	−10.403
**25**	−9.541	−10.366	−72.182	−10.366
**3**	−9.191	−10.232	−62.622	−10.232
**17**	−8.52	−10.194	−55.199	−10.194
**28**	−9.435	−10.132	−72.289	−10.132
**21**	−8.479	−10.128	−91.905	−10.128
**12**	−9.559	−9.935	−51.879	−9.935
**29**	−9.129	−9.825	−76.553	−9.825
**1**	−7.669	−9.8	−56.073	−9.8
**2**	−9.343	−9.753	−55.206	−9.753
**32**	−9.008	−9.704	−56.471	−9.704
**30**	−8.983	−9.679	−75.988	−9.679
**15**	−9.103	−9.624	−50.939	−9.624
**14**	−9.331	−9.492	−49.907	−9.492
**11**	−9.331	−9.492	−49.907	−9.492
**18**	−8.956	−9.485	−60.341	−9.485
**4**	−9.265	−9.341	−61.825	−9.341
**10**	−9.167	−9.328	−50.165	−9.328
**9**	−9.23	−9.31	−51.968	−9.31
**13**	−9.136	−9.216	−52.397	−9.216
**7**	−9.162	−9.162	−50.334	−9.162
**6**	−9.082	−9.154	−63.817	−9.154
**5**	−9.147	−9.147	−52.055	−9.147
**16**	−8.656	−9.106	−56.773	−9.106
**8**	−9.067	−9.068	−51.613	−9.068
**Y49_7BU4**	−9.049	−9.049	−93.921	−9.049

**Table 4:** Predicted in silico ADMET properties of the phenalenone derivatives.

Compounds *	Mol_MW	# Stars	Dipole	SASA	DonorHB	AccptHB	QPlogPo/w	QPlogS	QPlogKhsa	# Metab	QPlogBB	%Human Oral Absorption	QPlogHERG	CNS	# RtvFG
Recommended range	(130–725)	(0.0–5.0)	(1–12.50)	(300–1000)	(0–6)	(2.0–20.0)	(−2–6.5)	(−6.5–0.5)	(−1.5–1.5)	(1–8)	(−3–1.2)	(<25% poor; >80% high)	concern below −5	(−2 inactive) (+2 active)	(0–2)
**1**	287.271	0	2.269	482.741	4	5	0.637	−2.456	−0.357	6	−1.577	65.408	−3.892	−2	0
**2**	288.256	0	7.03	481.857	3	4.75	0.9	−2.549	−0.273	6	−1.552	67.526	−3.919	−2	0
**3**	358.347	0	4.081	543.911	2	5.75	1.679	−3.554	0.106	5	−1.509	71.209	−3.602	−2	0
**4**	404.373	0	9.43	616.737	3	10	0.76	−3.62	−0.273	3	−1.618	67.011	−4.155	−2	2
**5**	342.348	0	9.298	561.827	1	6	2.037	−3.9	0.109	3	−1.134	81.224	−3.898	−2	3
**6**	344.363	0	3.309	569.109	2	5.7	2.266	−3.979	0.158	4	−1.041	85.666	−3.946	−2	1
**7**	328.321	0	10.77	534.495	1	5.95	1.41	−3.29	−0.062	4	−1.567	69.494	−3.808	−2	2
**8**	364.782	1	12.714	596.584	1	5.95	2.161	−4.084	0.041	5	−1.884	72.456	−4.595	−2	2
**9**	330.337	0	7.925	528.447	1	4.5	2.59	−3.836	0.255	3	−0.728	91.237	−3.585	−1	1
**10**	300.31	0	5.524	498.414	1	3.75	2.366	−3.724	0.26	3	−0.796	86.825	−3.516	−1	0
**11**	300.31	0	5.616	500.081	1	3.75	2.398	−3.753	0.273	3	−0.795	87.024	−3.517	−1	0
**12**	300.31	0	9.368	548.255	1	3.75	2.634	−4.111	0.269	7	−1.29	85.236	−4.506	−2	0
**13**	314.337	0	7.36	523.476	0	3.75	3.022	3.848	0.268	3	−0.374	100	−3.613	0	0
**14**	300.31	0	5.617	499.932	1	3.75	2.399	−3.75	0.274	3	−0.794	87.031	−3.511	−1	0
**15**	344.32	1	13.714	566.499	0	6	1.573	−2.834	−0.308	6	−1.74	71.138	−4.202	−2	1
**16**	358.347	0	9.462	553.682	0	6	1.845	−2.591	−0.246	6	−1.417	76.489	−3.596	−2	1
**17**	330.293	1	15.038	518.961	1	6	0.984	−2.687	−0.224	6	−1.977	60.477	−3.804	−2	1
**18**	358.347	0	10.248	546.847	0	6	1.673	−2.299	−0.312	7	−1.588	72.711	−3.46	−2	1
**19**	594.744	2	9.484	789.214	4	7.45	5.322	−4.622	0.981	17	−2.174	57.778	−3.532	−2	0
**20**	610.743	7	9.975	1043.134	5	9.15	5.066	−7.89	0.907	17	−4.262	41.015	−6.435	−2	0
**21**	624.727	8	8.459	1026.611	5	9.45	5.31	−7.68	0.735	16	−4.35	28.338	−4.482	−2	1
**22**	610.743	7	9.879	1030.596	5	9.15	4.911	−7.677	0.918	17	−4.46	49.169	−6.255	−2	0
**23**	624.727	8	8.459	1026.616	5	9.45	5.31	−7.68	0.735	16	−4.351	28.338	−4.482	−2	1
**24**	652.78	8	12.335	1096.494	4	9.45	6.114	−8.908	1.288	17	−4.061	50.421	−6.561	−2	1
**25**	540.652	3	14.173	889.502	3	7.45	5.255	−6.976	0.997	13	−2.485	70.591	−5.556	−2	0
**26**	540.652	2	7.928	919.785	3	7.45	5.282	−7.502	1.064	13	−2.834	67.029	−5.832	−2	0
**27**	592.728	6	12.392	1005.377	3	7.45	6.358	−9.167	1.54	17	−2.88	73.816	−6.238	−2	0
**28**	592.728	6	11.434	1011.989	3	7.45	6.379	−9.284	1.561	17	−2.954	73.171	−6.288	−2	0
**29**	592.771	5	11.447	968.98	3	5.75	7.264	−8.949	1.821	13	−2.355	85.225	−5.777	−2	0
**30**	608.77	5	12.352	986.08	4	7.45	5.988	−8.302	1.398	14	−3.232	53.649	−5.838	−2	0
**31**	624.727	4	11.95	974.752	4	11.15	4.249	−7.089	0.71	14	−3.346	53.716	−5.828	−2	3
**32**	654.796	7	10.567	1030.237	3	10.85	5.564	−7.91	1.034	14	−3.042	68.666	−6.017	−2	1
**33**	606.711	5	9.825	992.894	3	8.75	5.513	−8.33	1.235	15	−3.349	62.369	−6.083	−2	1

* Recommended range: for 95% of known drugs; #Stars: # of descriptors that fall outside the 95% range of same values for known drugs. Large star number indicates less drug-likeness, and vice versa; Dipole: computed dipole moment; SASA: Total solvent accessible surface area; DonorHB: estimated number H^+^ to be donated in HB; AcceptHB: estimated number H^+^ to be accepted in HB; QLogPo/w: predicted octanol/water partition coefficient; QPlogS: Predicted aqueous solubility; QPlogKhsa: Prediction of binding to human serum albumin; #Metab: number of possible metabolic reactions; QPlogBB: Predicted brain/blood partition coefficient; % Human Oral Absorption: Predicted human oral absorption on 0 to 100% scale; QPlogHERG: Predicted IC_50_ value for blockage of HERG K^+^ channels; CNS: Predicted central nervous system activity; #RtvFG: Number of reactive functional groups.

## Data Availability

Not applicable.
